# A Spontaneous Missense Mutation in Branched Chain Keto Acid Dehydrogenase Kinase in the Rat Affects Both the Central and Peripheral Nervous Systems

**DOI:** 10.1371/journal.pone.0160447

**Published:** 2016-07-29

**Authors:** J. Samuel Zigler, Colin A. Hodgkinson, Megan Wright, Andrew Klise, Olof Sundin, Karl W. Broman, Fielding Hejtmancik, Hao Huang, Bonnie Patek, Yuri Sergeev, Stacey Hose, Cory Brayton, Jiao Xaiodong, David Vasquez, Nicholas Maragakis, Susumu Mori, David Goldman, Ahmet Hoke, Debasish Sinha

**Affiliations:** 1 Department of Ophthalmology, The Johns Hopkins University School of Medicine, Baltimore, MD, United States of America; 2 National Institute on Alcohol Abuse and Alcoholism, National Institutes of Health, Rockville, MD, United States of America; 3 Department of Neurology, The Johns Hopkins University School of Medicine, Baltimore, MD, United States of America; 4 Department of Biomedical Sciences, Texas Tech University Health Science Center, El Paso, TX, United States of America; 5 Department of Biostatistics & Informatics, University of Wisconsin School of Medicine and Public Health, Madison, WI, United States of America; 6 National Eye Institute, National Institutes of Health, Bethesda, MD, United States of America; 7 Department of Radiology, Children's Hospital of Philadelphia, Perelman School of Medicine, University of Pennsylvania, Philadelphia, PA, United States of America; 8 Department of Molecular and Comparative Pathobiology, The Johns Hopkins University School of Medicine, Baltimore, MD, United States of America; 9 Department of Neurosurgery, The Johns Hopkins University School of Medicine, Baltimore, MD, United States of America; 10 Department of Radiology, The Johns Hopkins University School of Medicine, Baltimore, MD, United States of America; University of Szeged, HUNGARY

## Abstract

A novel mutation, causing a phenotype we named *frogleg* because its most obvious characteristic is a severe splaying of the hind limbs, arose spontaneously in a colony of Sprague-Dawley rats. *Frogleg* is a complex phenotype that includes abnormalities in hind limb function, reduced brain weight with dilated ventricles and infertility. Using micro-satellite markers spanning the entire rat genome, the mutation was mapped to a region of rat chromosome 1 between *D1Rat131* and *D1Rat287*. Analysis of whole genome sequencing data within the linkage interval, identified a missense mutation in the branched-chain alpha-keto dehydrogenase kinase (*Bckdk*) gene. The protein encoded by *Bckdk* is an integral part of an enzyme complex located in the mitochondrial matrix of many tissues which regulates the levels of the branched-chain amino acids (BCAAs), leucine, isoleucine and valine. BCAAs are essential amino acids (not synthesized by the body), and circulating levels must be tightly regulated; levels that are too high or too low are both deleterious. BCKDK phosphorylates Ser293 of the E1α subunit of the BCKDH protein, which catalyzes the rate-limiting step in the catabolism of the BCAAs, inhibiting BCKDH and thereby, limiting breakdown of the BCAAs. In contrast, when Ser293 is not phosphorylated, BCKDH activity is unchecked and the levels of the BCAAs will decrease dramatically. The mutation is located within the kinase domain of *Bckdk* and is predicted to be damaging. Consistent with this, we show that in rats homozygous for the mutation, phosphorylation of BCKDH in the brain is markedly decreased relative to wild type or heterozygous littermates. Further, circulating levels of the BCAAs are reduced by 70–80% in animals homozygous for the mutation. The *frogleg* phenotype shares important characteristics with a previously described *Bckdk* knockout mouse and with human subjects with *Bckdk* mutations. In addition, we report novel data regarding peripheral neuropathy of the hind limbs.

## Introduction

It has been observed [1] that mutations affecting amino acid metabolism frequently have adverse neurological consequences. We report here a novel mutant which arose spontaneously in a colony of Sprague-Dawley rats. Animals homozygous for this mutation are characterized by splaying of the hindlimbs, decreased brain weight, and seizures, among other abnormalities. We show that this autosomal recessive phenotype, which we named *Frogleg*, arises due to a mutation in the gene *Bckdk*, which codes for the branched-chain keto-acid dehydrogenase kinase (BCKDK). Loss of BCKDK function results in neurological abnormalities in mice [2] and in a potentially treatable form of autism with epilepsy in humans [3]. BCKDK is an integral part of a mitochondrial enzyme complex, which regulates the levels of the branched-chain amino acids (BCAA), leucine, isoleucine and valine [4]. BCKDK regulates the activity of this catabolic pathway by phosphorylating, and thereby inactivating, BCKDH (branched-chain keto-acid dehydrogenase), the rate limiting enzyme in the pathway. The branched-chain amino acids are essential amino acids; i.e., they cannot be synthesized by the body, and their catabolism is highly regulated [5, 6]. If levels become too high, as happens if BCKDH is mutated and inactivated, a condition called maple syrup urine disease (MSUD) results [7]. MSUD has serious neurological effects, as does the converse situation where the levels of BCAAs are too low. Low BCAA levels may result from inactivation of BCKDK, such that the specific phosphorylation of BCKDH at Ser293 does not occur, thereby resulting in unchecked catabolism of the BCAAs and deficient circulating levels of the three amino acids [3]. The missense mutation reported here segregates with the *frogleg* phenotype, and correlates with markedly decreased phosphorylation of BCKDH at the critical residue Ser293 and with sharply decreased levels of plasma leucine, isoleucine and valine. In summary, the primary goal of this study, to identify the specific mutation responsible for the *frogleg* phenotype has been accomplished. Further study is required to fully understand the molecular mechanisms underlying specific aspects of the phenotype of this novel rat strain, *Bckdk*^*frogleg/frogleg*^.

## Results

### Initial breeding studies and genetic linkage analysis

Previously, we described a spontaneously occurring mutant (*Nuc1*) in the Sprague-Dawley rat, which affects various aspects of eye development and maps to the βA3/A1-crystallin gene at 10q25 [8,9,10]. A second *de novo* mutation arose in our *Nuc1* rat colony. Homozygotes for this phenotype exhibit a "frog-like" gait resulting from hind limb hypertonicity with hyperextension and dorsal rotation. Heterozygotes appeared to be completely normal. After failing to breed *frogleg* animals with each other or with wild type rats, non-affected littermates of the *frogleg* animals were randomly mated until pairings that resulted in affected progeny were identified. The two pairs found to produce *frogleg* progeny from this initial screening subsequently produced 7 litters with a total of 76 pups. Of these 76 progeny, 17 (22.4%) were phenotypically identified as *frogleg*, consistent with an autosomal recessive inheritance pattern. At this point all animals homozygous for the *frogleg* phenotype were also homozygous for *Nuc1*.

**Fig 1:**
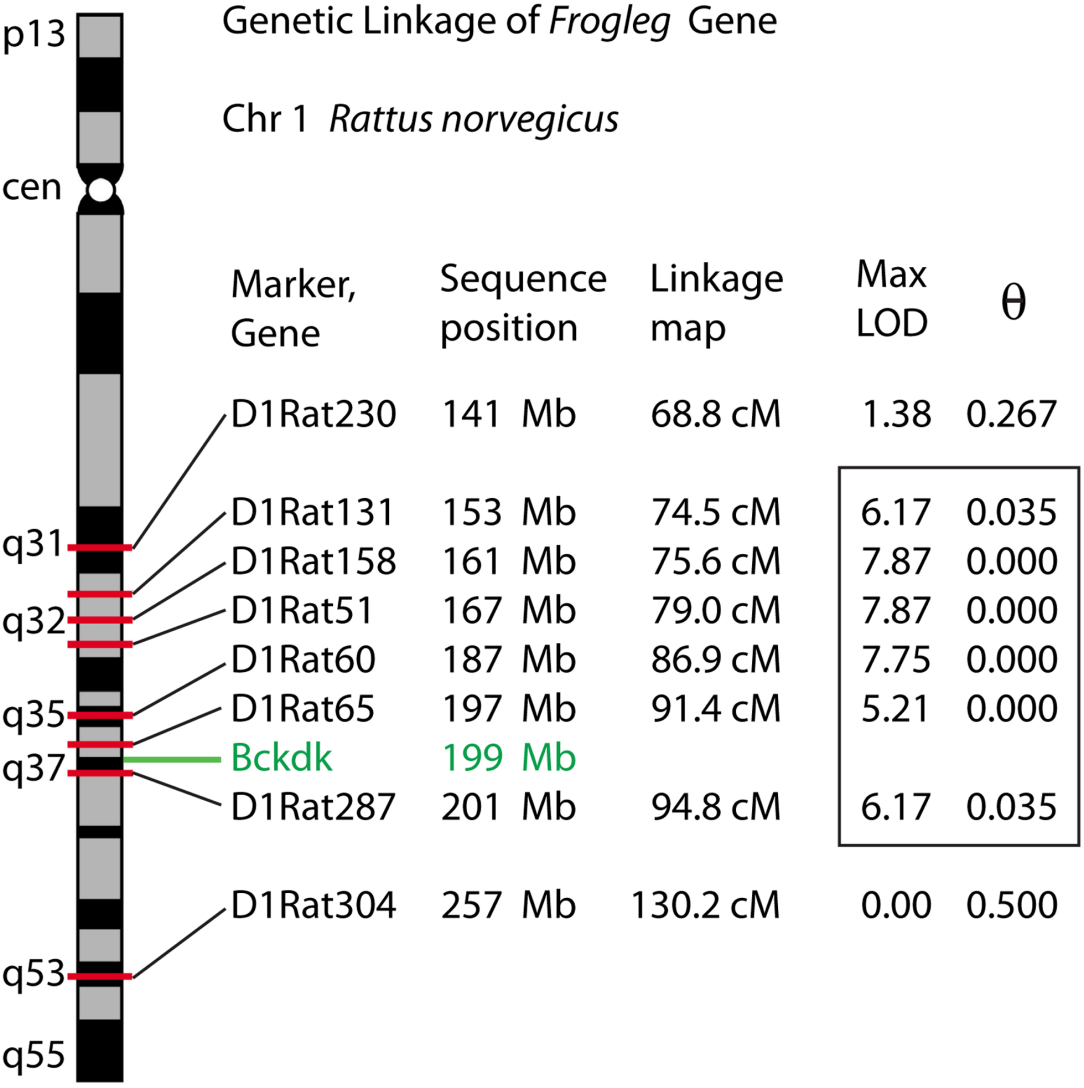
Linkage map of the *frogleg* locus on rat chromosome 1. Ideogram of rat chromosome 1, showing polymorphic markers in the region of the *Bckdk* gene. Nucleotide sequence positions were determined by locating original marker amplimer sequences to the RGSC 6.0 / rn6 July 2014 assembly of the rat genome. MLINK-derived maximum LOD scores and corresponding theta values indicate linkage distance to the disease locus. The analysis was based on 11 affected and 10 unaffected individuals. Significant linkage scores identifying the initial disease interval (> 3.0) are enclosed in the box.

Linkage was initially established to a region of rat chromosome 1 encompassing 1q32-1q37, covering approximately 50 Mb and containing about 175 genes. A maximum LOD score of 7.87 was obtained in the interval between markers, *D1Rat131* and *D1Rat287* (Fig 1). Additional F2 and F3 individuals were genotyped. This identified *D1Arb18*, a marker that co-segregated with the mutation in all crosses, and later proved to be less than 1 Mb up from the locus. Analysis of whole genome sequence (WGS) data within the linkage interval (159,182,239–208,389,496 genome build rn5) identified 3002 homozygous variants in the *frogleg* mutant not seen in the wild type littermate. Only 98 variants altered coding sequences (22), splice site (13), or conserved promoter elements (63). The remainder were intronic, intergenic, or within the untranslated portions of transcripts. Included among the missense variants was the G369E variant in the *Bckdk* gene which was predicted to be highly damaging using PROVEAN [11], with a score of -7.477, using a cutoff of lower than -2.5 for damaging. There were no other missense mutations in *Bckdk*. No other novel missense variant for which analysis was possible was predicted to be damaging. A complete list of homozygous variants found in the *frogleg* interval is provided in S1 Table. A list of the putative functional variants and their possible consequences is shown in S2 Table.

**Fig 2:**
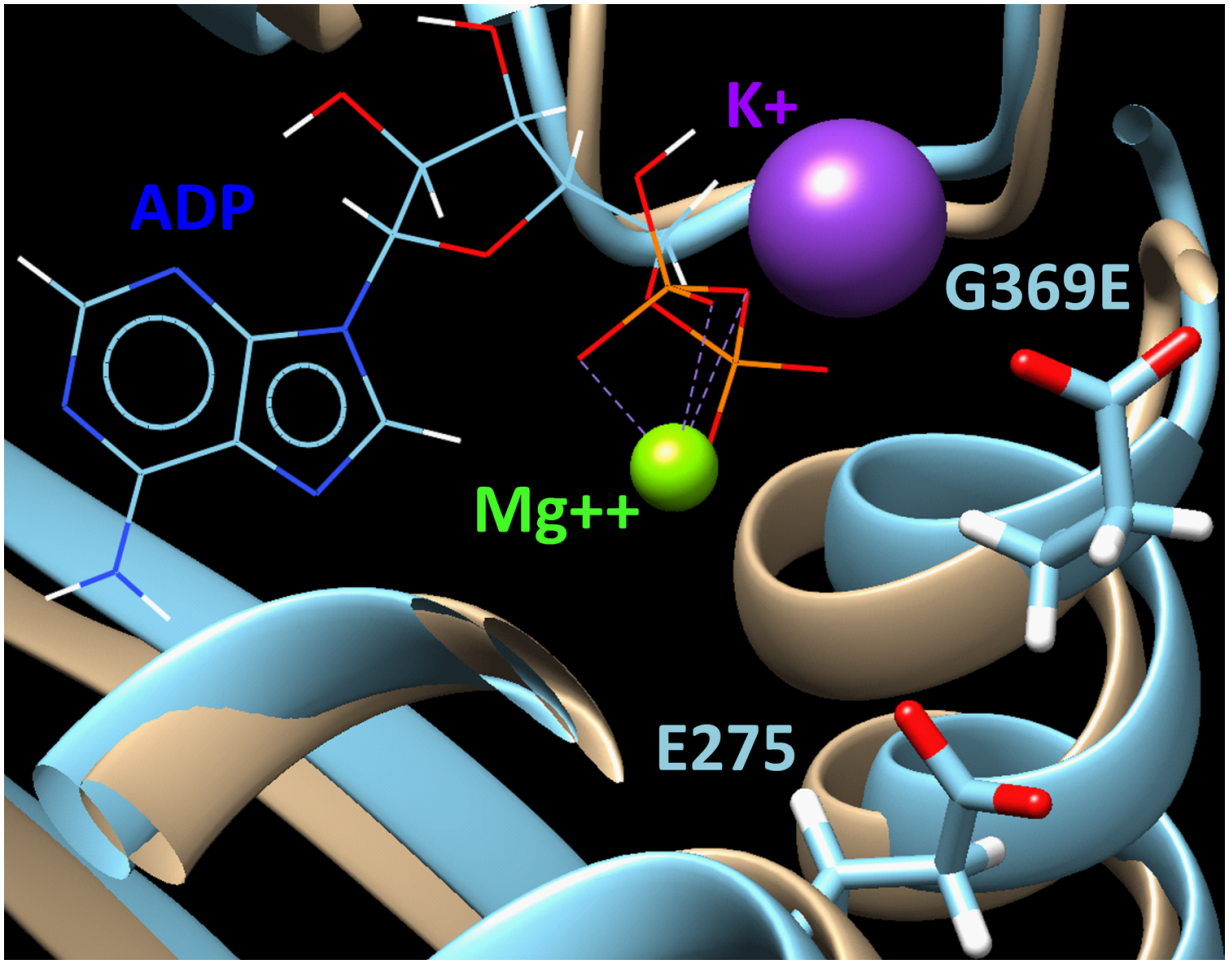
The effect of the G369E mutation on the structure of BCKDK protein is shown. The prediction is based on homology modeling of the structures of BCKDK and the G369E mutant as refined using the 7.5 ns molecular dynamics and shown in beige and light blue, respectively. In the mutant, the helix containing residue E369 is moving outward from the ADP binding site due to repulsion from the negatively charged residue E275. This might suggest a decrease in ADP binding activity by the mutant enzyme due to the replacement of glycine in normal BCKDK by the negatively charged glutamic acid in the mutant variant.

The non-conservative *Bckdk* G369E amino acid substitution is found at an extremely conserved site in the *Bckdk* gene. All vertebrate species examined including *Homo sapiens*, *Mus musculus*, *Falco peregrinus* (bird), *Xenopus tropicalis* (frog) and *Danio rerio* (fish) encode a *Bckdk* orthologue with a glycine at the homologous position, surrounded by a block of invariant amino acids. While *Bckdk* orthologous genes in the invertebrates *Strongylocentrotus purpuratus* (sea urchin), *Aplysia californica* (molllusk), *Drosophila melanogaster* (insect), *Caenorhabditis elegans* (nematode), and *Hydra vulgaris* (coelenterate) reveal more sequence divergence in this portion of the protein, the G369-homologous glycine and neighboring amino acids are completely conserved. Based on molecular dynamics modeling (Fig 2), the G369E mutation is predicted to have an effect on the structure of the highly conserved serine protein kinase domain.

### BCKDK deficiency

**Fig 3:**
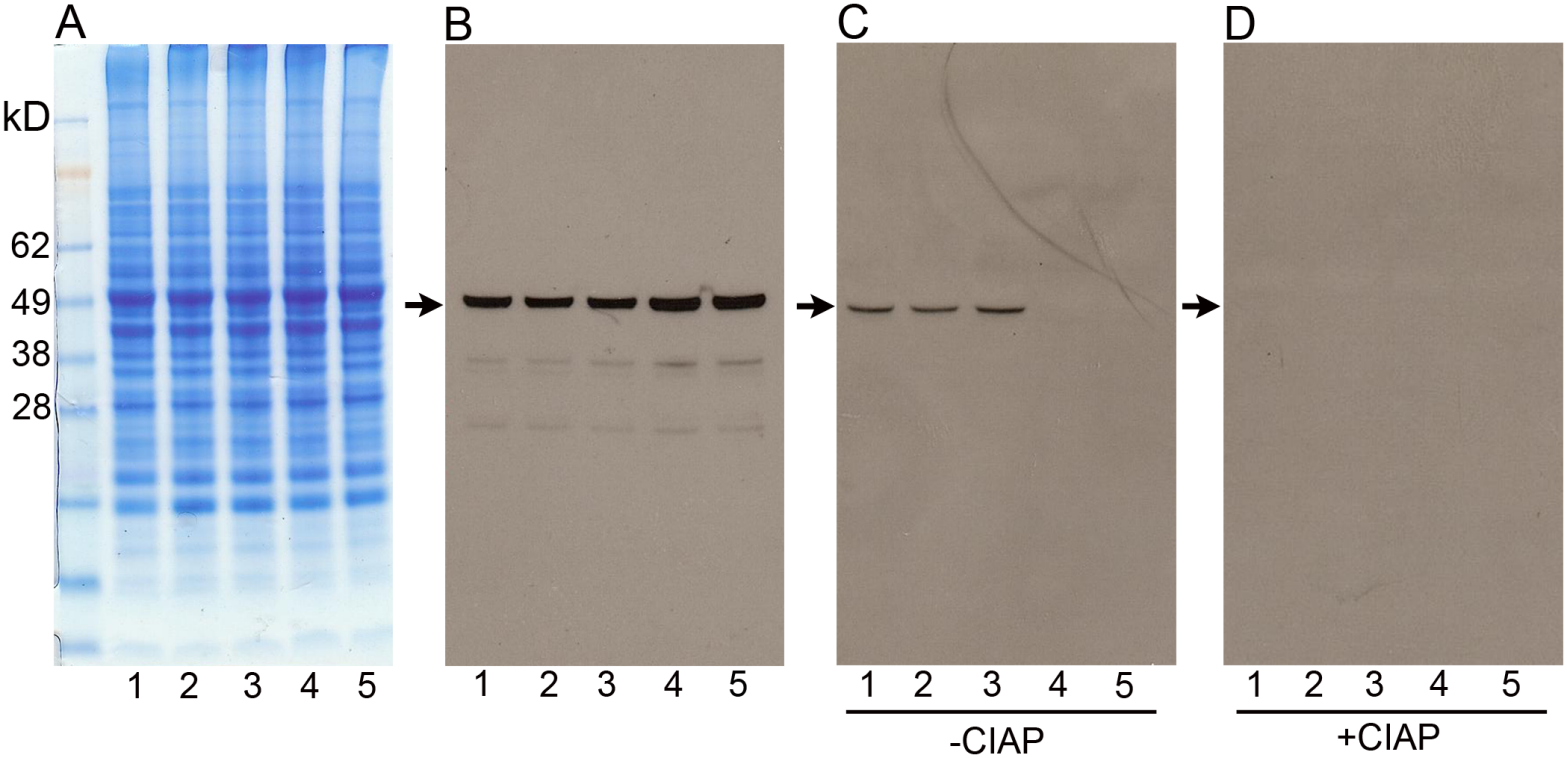
Electrophoresis and western analysis of protein lysates from the brains of *frogleg* and littermate rats. In panel A are shown the staining patterns for 5 individual rats: lane 1 is lysate from a wild type, lanes 2 and 3 are from rats heterozygous for the *Bckdk* mutation, and lanes 4 and 5 are from homozygotes. In panel B equal aliquots of the same 5 samples were electrophoresed, transferred to nitrocellulose and probed with an antibody to BCKDH subunit E1α. It is evident that similar amounts of the total enzyme are present in all 5 samples. In panel C the amount of BCKDH phosphorylated at Ser293 of the E1α subunit is determined by probing the same 5 lysates with an antibody specific for the phosphorylated form of the enzyme. While the wildtype and heterozygous animals have a clear band, no immunoreactivity is detected in the 2 samples homozygous for the mutation. To demonstrate that the bands present on panel C represent the phosphorylated enzyme, aliquots of the same lysates were treated with calf intestinal alkaline phosphatase before electrophoresis. The bands present in lanes 1–3 of panel C are no longer visible.

In order to establish that the mutation in *Bckdk* is responsible for the *frogleg* phenotype, we first used western blotting to determine the phosphorylation state of its target protein, BCKDH. Animals from the same litter that were phenotypically *frogleg*, or had been genotyped as wild type or as heterozygotes for *frogleg*, were euthanized and brain extracts prepared as described in Methods. Fig 3A shows the Coomassie blue staining patterns for representative extracts. In panel 3B, identical aliquots of the same homogenates were electrophoresed, transferred to nitrocellulose and probed with an antibody directed against the E1α subunit of BCKDH. All 5 samples have similar amounts of total BCKDH (predicted band size ~50kD) based on immunoreactivity. To ascertain the phosphorylation status of BCKDH in the various samples, another aliquot of the supernatant (with phosphatase inhibitors) was run, transferred, and probed with an antibody specific for BCKDH E1α subunit phosphorylated at Ser293 (Fig 3C), the specific phosphorylation catalyzed by BCKDK. A clear band at the expected size is present in the wild type and heterozygote samples, but is not visible in either of the two *frogleg* samples. To definitively prove that these bands represent the phosphorylated enzyme, equal aliquots were taken from the portion of the original homogenates not protected with phosphatase inhibitors and were incubated with calf intestinal alkaline phosphatase (CIAP) as described in Methods. When run on the same gel and processed like the samples in Fig 3C, the bands present on the first 3 lanes of panel C were absent following phosphatase treatment (Fig 3D). To confirm that increased catabolism of the branched-chain amino acids was occurring in *frogleg*, as would be expected since phosphorylation at Ser293 was deficient, complete amino acid analyses were conducted on plasma samples from *frogleg*, wild type and heterozygous rats. Table 1 shows the data for the 3 branched chain amino acids with a 70–80% decrease in each case in *frogleg*, as compared to wildtype. No significant difference was found for any of the other amino acids. Levels of the branched-chain amino acids were marginally lower in the heterozygous animals than in wild type littermates (Table 1). Complete amino acid analysis data are provided as S3 Table.

**Table 1:** 

Branched-chain Amino Acid Levels in Plasma (μmoles/liter ± S.D.)			
	Wild type	Heterozygote	*Frogleg*
**leucine**	131.8 ± 8.9	110.7 ± 11.4	30.2 ± 8.5
**isoleucine**	57.0 ± 5.2	48.7 ± 7.4	10.8 ± 2.9
**valine**	132.0 ± 12.9	114.0 ± 18.6	39.0 ± 7.2

### Growth of *Frogleg* rats

**Fig 4:**
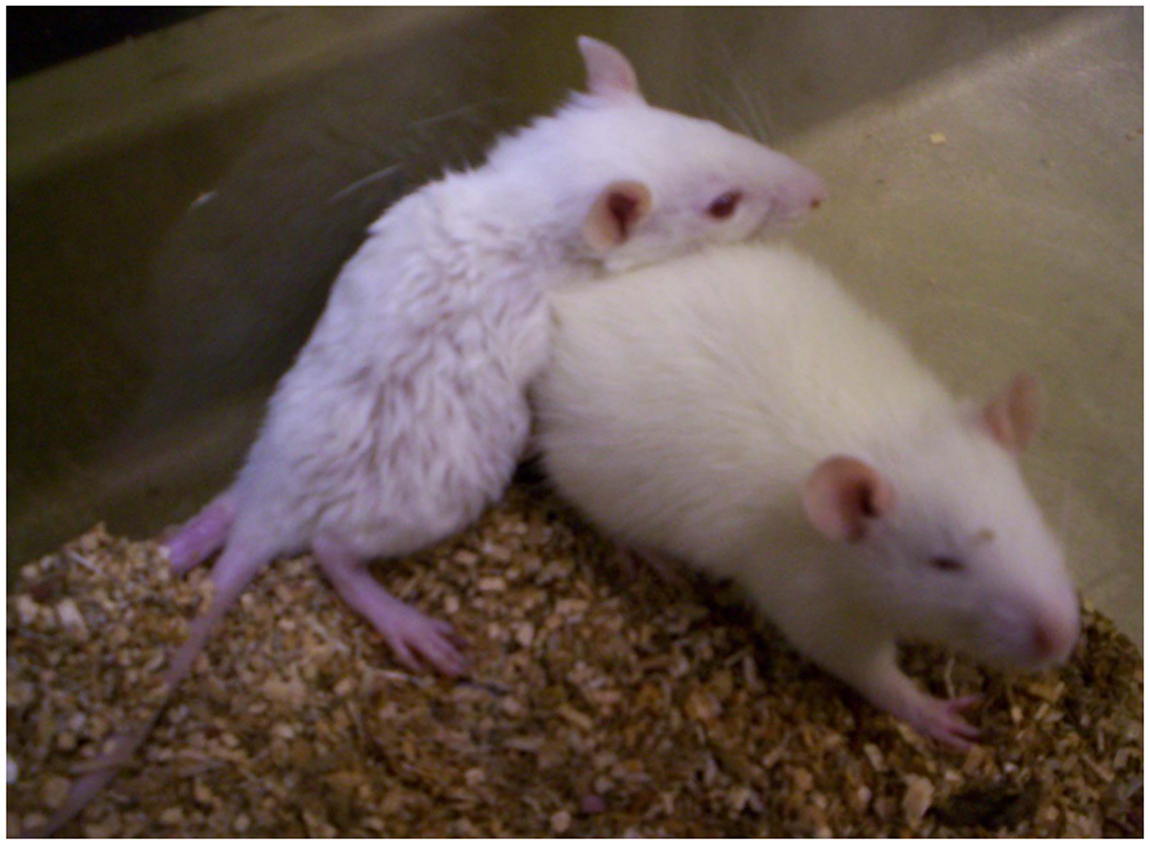
Appearance of *frogleg* homozygous rat at 20 days of age. The animal on the left shows the typical appearance of the *frogleg* pup, relative to a non-*frogleg* littermate on the right. Note the small size, the thin, rough coat, and the abnormal rotation of the hind limbs of the affected animal.

**Fig 5:**
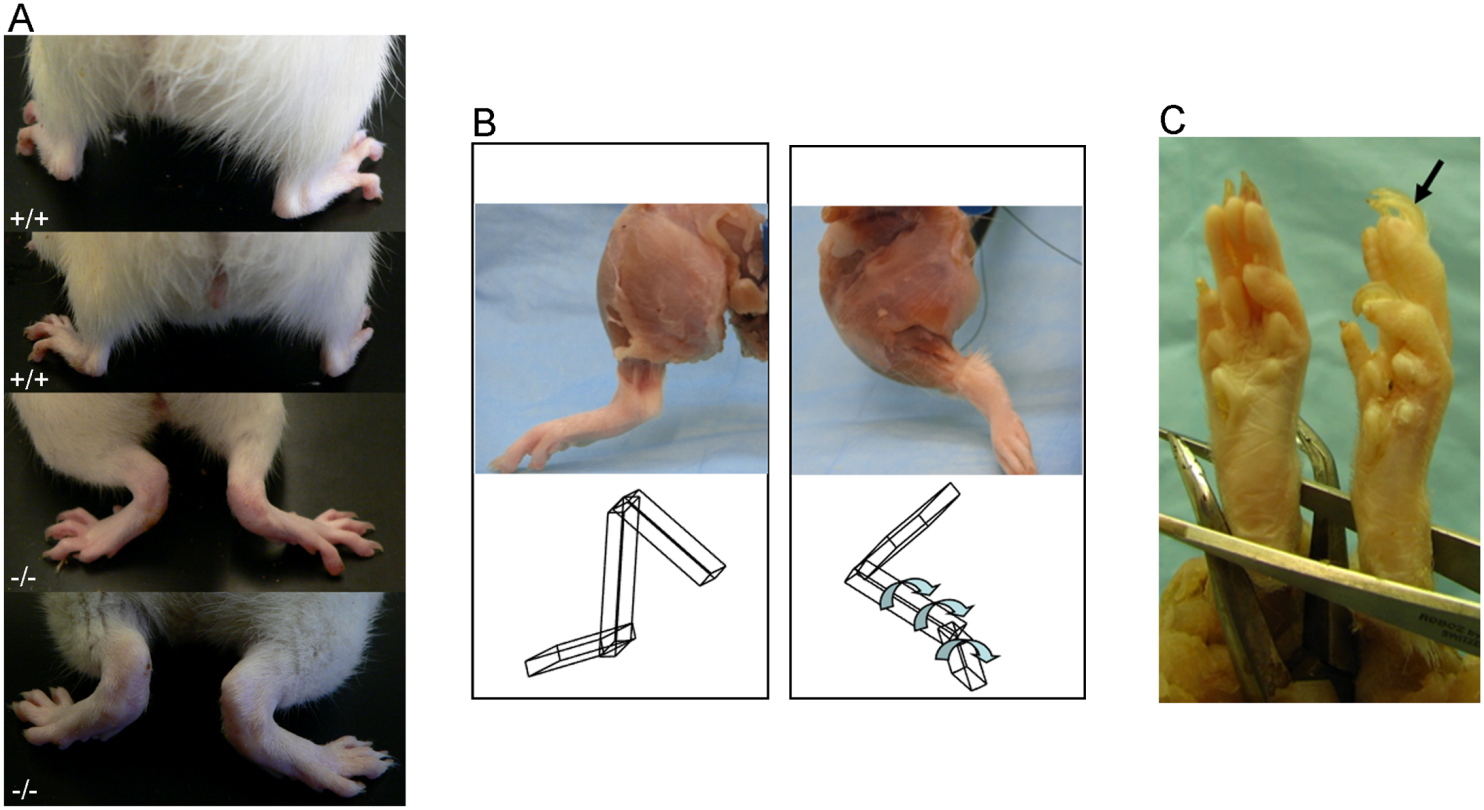
A) Impairment of the hind limbs of *frogleg* rats (bottom two panels) compared to wild type Sprague Dawley rats at 4 months (top two panels). B) Left hind limbs of a wildtype (left) and a *frogleg* (right) rat. Note the inner rotation and extension of the *frogleg* rat compared with the normal alignment and orientation of the wild type. Also, note the similarity in muscle mass. The cartoon at the bottom of the figure demonstrates the joint alignment and orientation. C) The hind paw of a wild type and a *frogleg* rat are shown in (left) and (right), respectively. Note the in-turning (pronated) ankles in *frogleg* with hyperkeratosis of foot pads and toenail overgrowth (arrow), likely related to abnormal or reduced wear.

The characteristic hind limb abnormality of the *frogleg* animals becomes apparent as soon as the pups begin moving around the cage (S1 Video), usually at about 12–14 days of age. By the same age affected pups are typically smaller than their unaffected littermates and have a rough, thin coat as seen in Fig 4, where a 20 day old *frogleg* animal (left) is shown with a non-*frogleg* littermate. To compare the rates of growth of affected and normal pups, we weighed animals in six different litters born to *frogleg* heterozygote parents starting at 1 day of age and continuing for 10 weeks. Comparisons were made only within litters, because litter size strongly affects growth rate. In each litter studied there was no difference in average weight of the pups during the first week of life, but at day 7 or 8, weights for the *frogleg* animals began to diverge from their littermates, a trend that continued for 2–3 weeks when the *frogleg* animals averaged about 30% lower in body weight (about 35–45 grams as compared to 55–65 grams for the normal littermates). For the next 4–5 weeks both groups of pups grew at a similar rate, maintaining the approximately 20 grams deficit for the affected animals. Subsequently, the deficit gradually diminished and by about 10 weeks of age the body weights of the affected animals were not different from their normal littermates. Also, by about ten weeks of age, the coats of the affected animals no longer were thin and rough, but had a normal appearance. The abnormalities of stance and gait, however, remained unchanged. *Frogleg* rats also have splayed hind limbs (Fig 5A), abnormal orientation of the hind limbs with inner rotation and extension (Fig 5B), and show pronation of ankles and overgrowth of claws (Fig 5C). The images shown are representative of the entire population of rats homozygous for the mutation that were examined. While observations were made on both awake and anesthetized animals, we did not perform measurements to quantify the defects. We can say that internal rotation was greater than 80 degrees of rotation and can be classified as severe.

### General Pathological Evaluation

**Fig 6:**
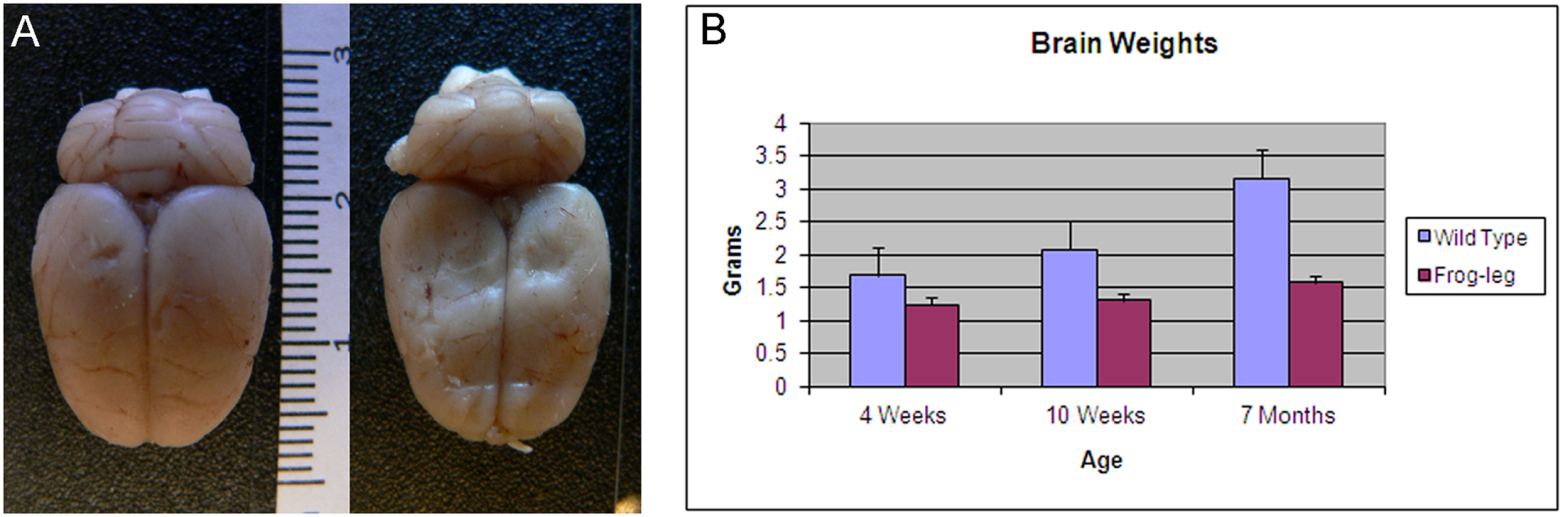
**A**. Brains from wild type (left) and *frogleg* (right) were of similar size. Scale is in centimeters. **B**. Brain weights, however, differed in wild type (+/+) and homozygote (*frogleg*) rats of 4 weeks, 10 weeks and 7 months of age, being considerably smaller in the *frogleg* rats compared to their littermates at all ages tested (mean+SEM).

**Fig 7:**
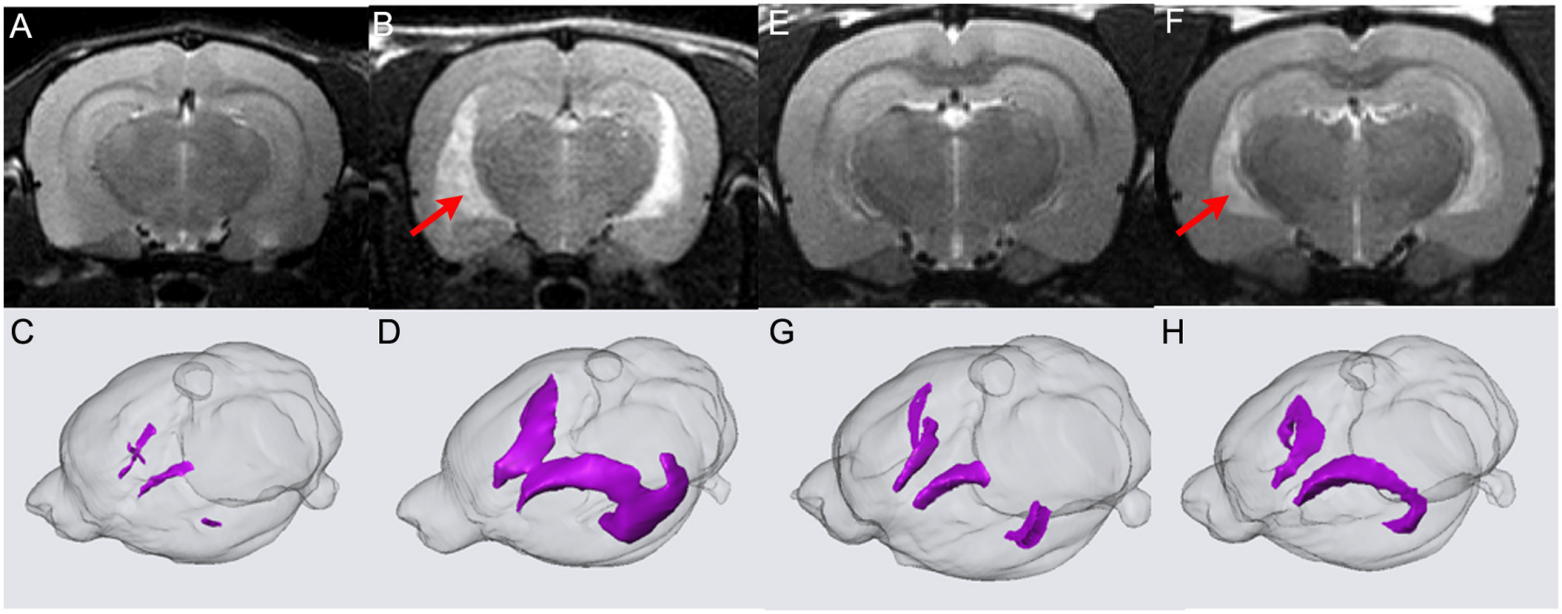
2D coronal T2 weighted images of representative wild type **(A)** and f*rogleg* **(B)** brains at P65. In **(C)** and **(D)** 3D reconstructions of the P65 lateral ventricles are shown. Dramatic difference in ventricular size is clearly observed. In **(E)** and **(F)** 2D coronal T2 weighted images of wild type and *frogleg* brains, respectively, are shown at P110. In **(G)** and **(H)** the 3D reconstructions for P110 are shown. The difference of ventricular size is not as great as that shown in **(C)** and (**D)**. Red arrows **(B, F)** indicate the enlarged ventricles of *frogleg* brains.

**Fig 8:**
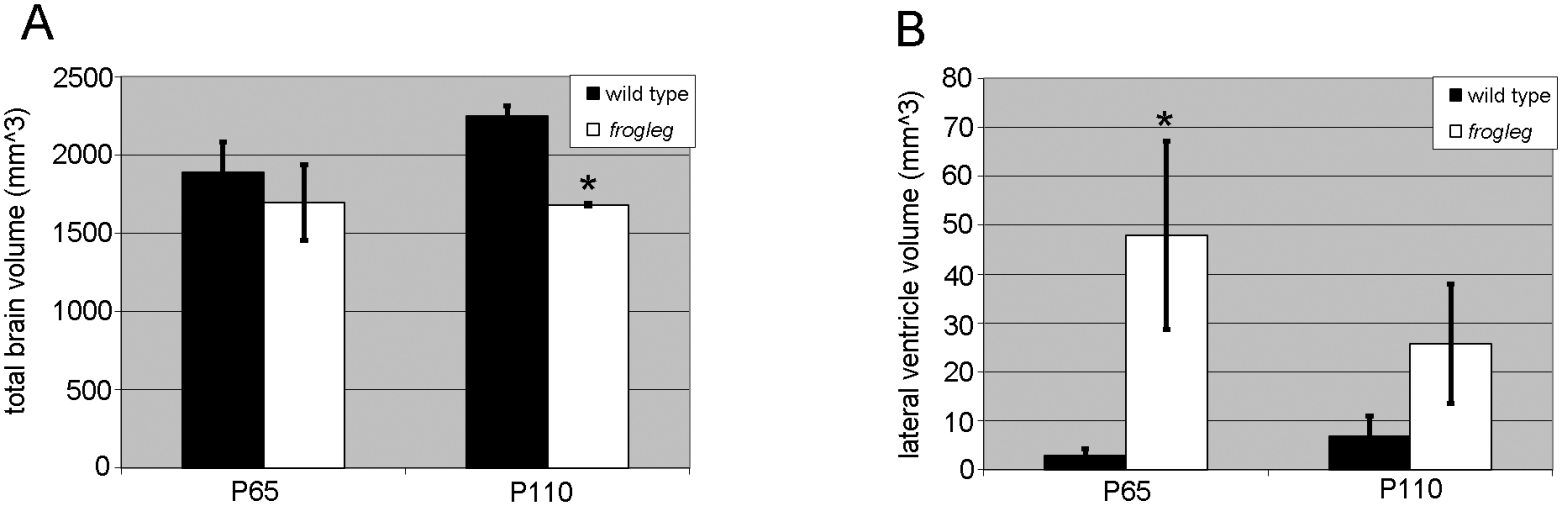
**A.** Averaged total brain volume of wild type and *frogleg* rats at P65 and P110; **B.** Averaged ventricular size of wild type and *frogleg* rats at P65 and P110. Error bars represent SD. * P< 0.05 (relative to wild type).

After x-ray and Magnetic Resonance Imaging (MRI) studies on the hind limbs revealed no structural abnormalities in the *frogleg* animals when compared to wild type littermates, we turned our attention to possible neurological bases for the phenotype. While the appearance and size of the brains were similar, the weights of the brains from affected animals were significantly lower than those of unaffected littermates (Fig 6A & 6B). At 10 weeks of age, when body weights of the two groups had equalized, the brain weight in the *frogleg* group was only about 65% that of the unaffected animals. This deficit in weight continued to increase, reaching about 50% of normal brain weight, by 7 months of age. At least a portion of the reduced brain weights in the *frogleg* animals can be accounted for by ventricular dilation as determined by *in vivo* MRI. In Fig 7, 2D coronal T2 weighted images for brains of representative wild type and *frogleg* animals at 65 and 110 days of age are shown in the upper images, with 3D reconstructions of the lateral ventricle for each brain shown below. Arrows indicate the enlarged ventricles in the *frogleg* brains. Fig 8A compares total brain volume for the various groups. Consistent with the brain weight data, the brain volume of the *frogleg* animals changes little as age increases. The marked increase in the size of the ventricles in the affected animals is shown graphically in Fig 8B.

**Fig 9:**
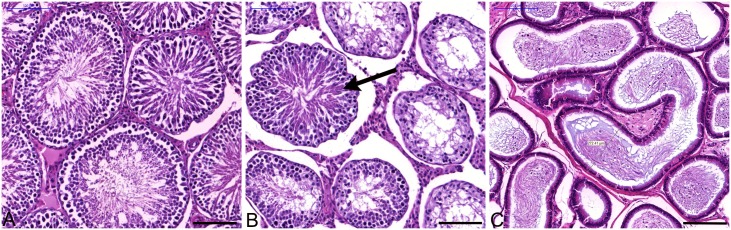
Wild type and *frogleg* testes and epididymis, paraffin, 5μ sections, H and E. A. Wild type testis, with normal spermatogenesis. B. *Frogleg* testis, multiple seminiferous tubules with no spermatogenesis; only 1 tubule (arrow) with active spermatogenesis. C. *Frogleg* epididymis, reduced sperm (oligospermia) with clumped and degenerating cells. Scale bars = 100μm in A and B; 200μm in C.

To obtain a more global picture of the *frogleg* phenotype, two *frogleg* rats (1 female 3.5 months old, 1 male, 5.5 months old), two phenotypically wild type littermates, and two purchased Sprague- Dawley (SD) rats were submitted for complete necropsy. On survey radiographs, spine and limb anatomy of *frogleg*, wild type and SD rats were similar and considered to be within normal limits. Limbs and musculature were similar on appearance and palpation, but *frogleg* rats were identified easily by their stance and gait. Substantive differences were not identified in CNS or peripheral nervous system (PNS) of *frogleg* or wild type rats in these immersion fixed, paraffin embedded survey specimens. Mild changes found in particular animals (*frogleg* or normal), and considered to be incidental, included small thymic cysts, mild mineralization in the kidney tubules, hemosiderin in the spleen, mild vacuolation in CNS white matter, and scattered dark neurons in CNS. Examination of multiple *frogleg* brain sections showed normally formed neocortex, hippocampus, basal ganglia, thalamus, brainstem, and cerebellum. However, dilated ventricles were identified in these *frogleg* brains, consistent with MRI findings. Toluidine-blue stained epon sections of *frogleg* nerve, revealed some abnormal Schwann cells with distended cytoplasm, granular inclusion material, and enlarged prominent nucleoli. Hind limb musculature of *frogleg* animals had scattered, shrunken and angular myofibers suggestive of a denervation atrophy. The male *frogleg* rat had smaller testes than the wild type and SD rats, and histopathology revealed oligospermia with reduced and abnormal seminiferous epithelium and spermatogenesis, consistent with the clinical observation of sub-fertility (Fig 9). In addition, when suspended by the tail, *frogleg* rats showed clinching of the hind limbs to the body (S2 Video).

### Electrophysiological Analysis

**Fig 10:**
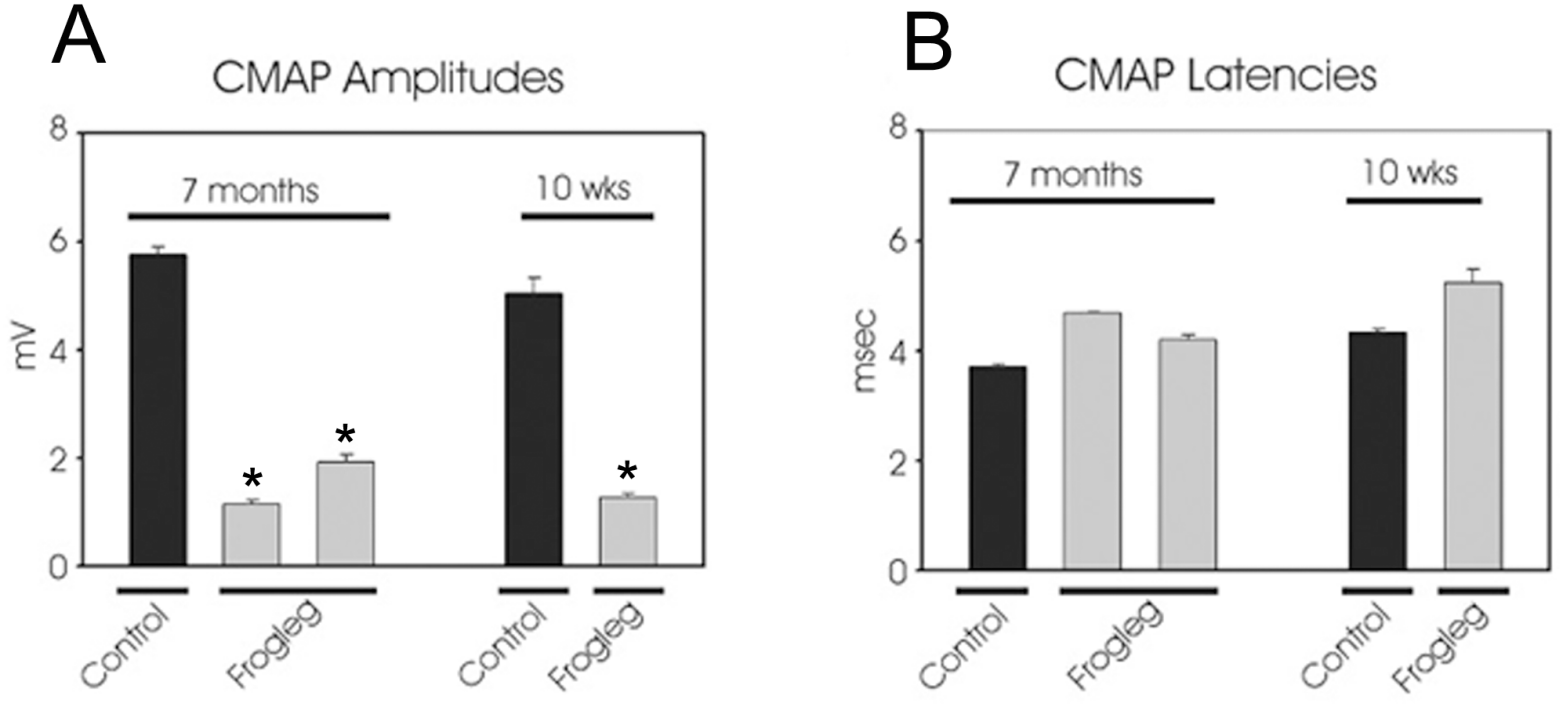
**A.** Sciatic compound muscle action potentials (CMAP) are markedly reduced in two representative 7 month old *frogleg* rats when compared with an age-matched control (left panel). A similar decrease in CMAP was also evident in *frogleg* rats at 10 weeks of age (right). **B.** CMAP latencies at both 7 months and 10 weeks of age were marginally higher in *frogleg* rats. Error bars represent SEM. * P< 0.05 (relative to control).

Sciatic compound muscle action potentials (CMAP) were determined for *frogleg* and age-matched control animals at 10 weeks and 7 months of age by nerve conduction study. As seen in Fig 10A, CMAP amplitudes for *frogleg* animals were between 1 and 2 mV at both ages tested, while control animals of both ages had amplitudes between 5 and 6 mV. CMAP latencies were marginally increased in the *frogleg* animals (Fig 10B). These data reflect a significant axon loss in the sciatic nerves of the *frogleg* animals.

### Transmission Electron Microscopy (TEM) analysis of the sciatic and sural nerves

**Fig 11:**
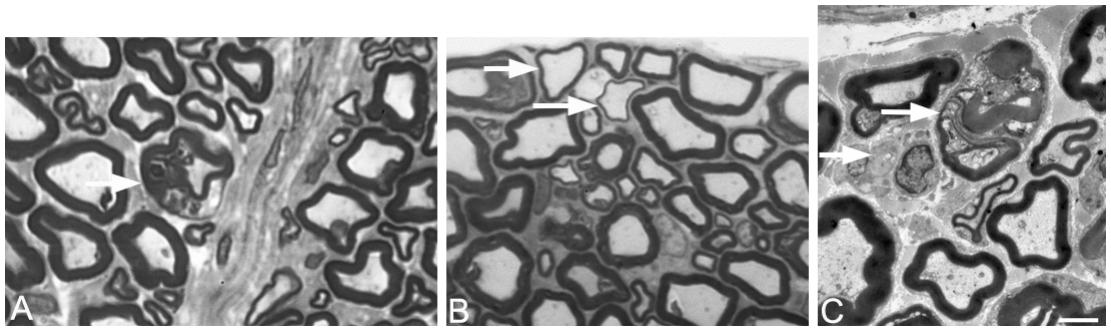
Transmission electron microscopy images of cross sections of hind limb nerves from a *frogleg* rat. A. Sciatic nerve showing evidence of rare active demyelination (arrow). In the ventral root (B) of the sciatic nerve (as well as in the nerve itself) there were axons that were thinly myelinated (arrows in B). In the sural nerve, there was evidence of rare Wallerian-like degeneration, intra-axonal inclusions, and denervated Schwann cells. Scale bar = 2 microns.

To determine whether motor pathways, sensory pathways, or both, are affected in the *frogleg* rat, we conducted TEM analyses on the sciatic and sural nerves and on the L4 and L5 ventral roots for motor neurons and the L4 and L5 dorsal roots for sensory neurons. Rare active demyelination was observed in the sciatic nerve; thinly myelinated axons were present in both the sciatic nerve (Fig 11A) and the ventral root (Fig 11B). The sural nerve (sensory) was also abnormal with evidence of rare Wallerian-like degeneration, denervated Schwann cells, and intra-axonal inclusions (Fig 11C). It should be noted that in all these cases only 1–2% of axons were obviously affected.

### Analysis of neuromuscular junctions

**Fig 12:**
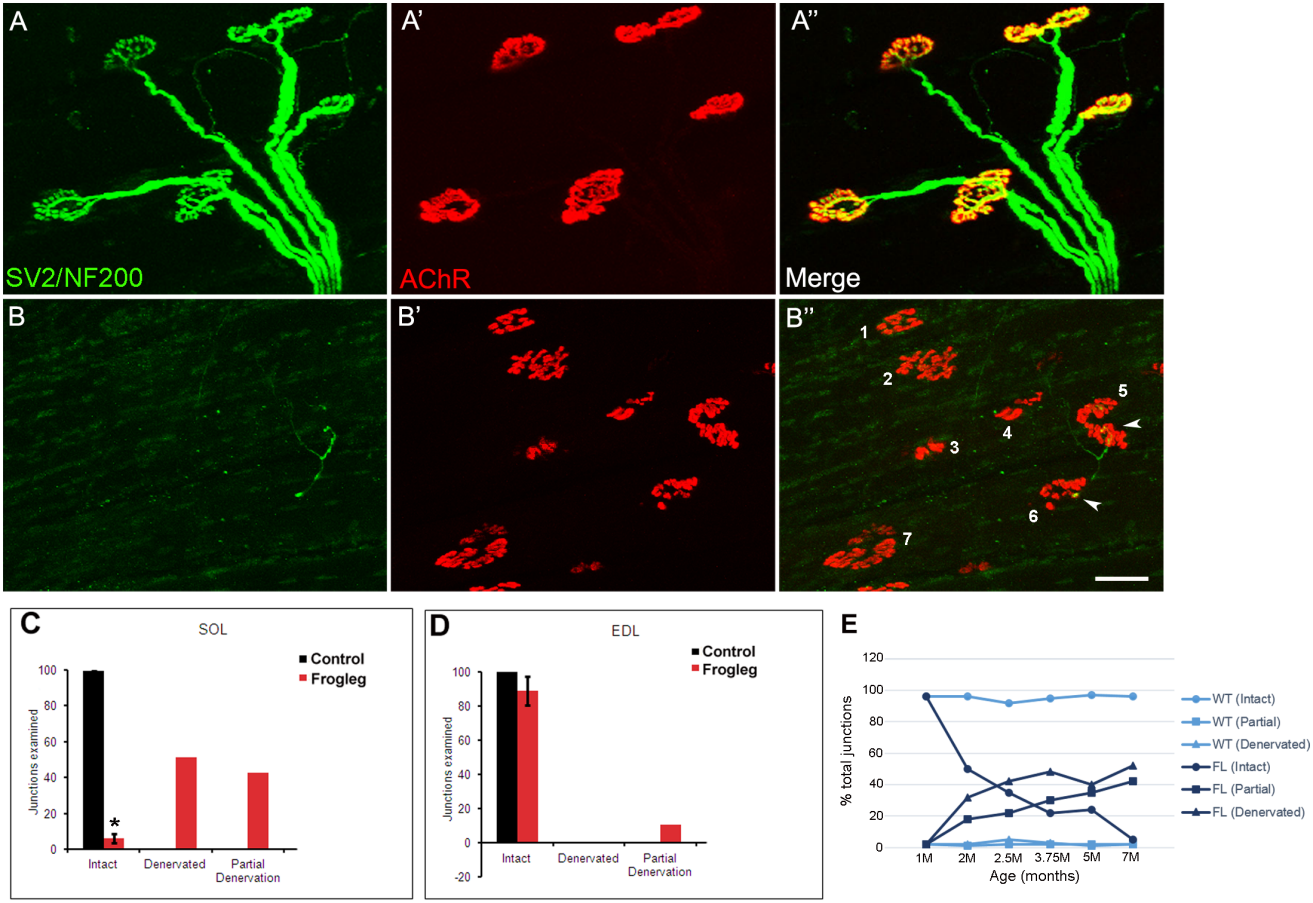
Immunolabeling of neuromuscular junctions of soleus and extensor digitorum longus (EDL) muscles. Representative data for muscles isolated from 7 month old *frogleg* rat are shown in Panels A for the EDL and Panels B for the soleus. For the EDL, panel A shows staining (green) with antibodies to neurofilament (NF200) and synaptic vesicle protein (SV2), while panel A’ shows staining of the nicotinic acetylcholine receptors at the junction with α-bungarotoxin (red). A” shows the merged image, with yellow indicating normal innervation of each of the 5 junctions shown. In contrast, for the soleus, panel B indicates very little immunoreactivity with SV2 or NF200. Staining of the nicotinic acetylcholine receptors in B’ is normal, but in the merged image (B”) only 2 of the 7 numbered junctions show evidence of partial innervation (arrowheads at numbers 5 and 6), with the other 5 being fully denervated. Scale bar = 50 μm. Panels C and D show the cumulative data for soleus and EDL muscles respectively, for 7 month old animals. Black bars are for wild type and red bars for *frogleg* animals. In WT EDL and soleus muscles, 97.1% and 98.6% of NMJs are intact. In contrast, while the EDL NMJs were 89.8% intact in the *frogleg* mutant at 7M, only 4.4% of the NMJs for the soleus remained intact at that age. Signs of denervation were predominant in soleus muscles, with NMJs being either partially (42.6%) or completely (51.1%) denervated. Error bars = SD; * P< 0.05 (Relative to control). In panel E, the time course of denervation in the soleus muscle for *frogleg* and wild type rats aged 1–7 months is shown.

To determine the status of neuromuscular junctions in the hindlimb of the *frogleg* rat, the soleus (slow-twitch, postural) muscle and the extensor digitorum longus (EDL, fast twitch) muscle from *frogleg* and normal rats of various ages were analyzed. Representative data are shown in Fig 12 for muscles taken from 7 month old animals. In A, the EDL muscle from a *frogleg* rat is immunolabeled (green) with the antibodies for neurofilament (SMI312) and synaptic vesicle protein (SV2). In A’, α-bungarotoxin labeling (red) of the same section shows strong staining of the nicotinic acetylcholine receptors at the neuromuscular junctions. In the merged image (A”) there is clear co-localization of the labels at the neuromuscular junctions and complete innervation of each of the 5 junctions present. In contrast, analyses of the soleus muscle from 7 month old *frogleg* rats revealed minimal evidence for immunoreactivity with either SV2 or SMI312 (B), although staining of the nicotinic acetylcholine receptors (B’) was similar to that shown for the EDL (A’) as well as muscles of wild type rats. The merged image (B”) shows only very limited co-localization of the labels at 2 of the 7 junctions shown (#5 and 6, arrowheads), with very little evidence of junction innervation. Panels C and D summarize these data for the soleus and EDL muscles, respectively, showing the percentages of intact, denervated and partially denervated junctions in wild type and *frogleg* rats at 7 months of age. When these studies were repeated on muscles from 1 month old rats, there was essentially no difference found between the normal and *frogleg* rats. Subsequently, analyses were performed at several intermediate ages to establish a time course for the loss of innervation of the soleus muscle in *frogleg* (panel E). Time course data are not shown for the EDL since loss of innervation was so little at 7 months.

## Discussion

The catabolism of BCAAs is effected by a mitochondrial matrix enzyme complex that is tightly regulated to maintain optimal levels of leucine, isoleucine and valine. BCKDK is a kinase integral to this complex that specifically phosphorylates the E1α subunit of BCKDH at residue Ser293. BCKDH catalyzes the rate-limiting step of this critical metabolic pathway [12] and when phosphorylated, its activity is inhibited. When not phosphorylated, activity is unchecked and levels of the BCAAs become depleted. We identified a missense mutation segregating with the *frogleg* phenotype in the *Bckdk* gene, resulting in a glycine to glutamic acid conversion at residue 369 of BCKDK. G369 is in the kinase domain of BCKDK and is a very highly conserved residue across a wide phylogenetic range, including all metazoans [12]. The glycine to glutamic acid change has a PROVEAN score of -7.477, indicative of a damaging effect [11]. To demonstrate that this variant has functional significance, we have assessed the level of phosphorylation of BCKDH in *frogleg* and unaffected littermate rats. The specific phosphorylation at Ser293 does not occur at significant levels in the *frogleg* animals. In addition, relative to wild type and heterozygous littermates, plasma amino acid levels of the BCAAs are greatly reduced in the *frogleg* animals. This would be expected, since in the absence of phosphorylation at Ser293 the activity of BCKDH, and the catabolism of the branched-chain amino acids, is unchecked. Moreover, comparison of the *frogleg* phenotype with that of a mouse line in which *Bckdk* was knocked out [2] and with characteristics reported for human patients with mutations in *Bckdk* [3], further supports the conclusion that the G369E change causes the *frogleg* phenotype.

The most obvious of the phenotypic abnormalities in *frogleg*, the defect in hind limb function, becomes apparent during the second week after birth, as soon as the animals begin to move around the cage. In affected animals a decreased rate of growth is consistently detectable by postnatal day 7 or 8, and the presence of a thin, rough coat is typically appreciated as soon as the coat develops in the unaffected littermates. While both the growth and coat abnormalities resolve by the third month of life, the hind limb defect does not, although it does not appear to be progressive within the age range (~1 year) that we have kept *frogleg* animals. The *Bckdk* knockout mouse was shown to have a similar array of signs including abnormal gait with hind limb splaying, hind limb flexion throughout life, delayed growth, and fur abnormalities. Another characteristic which has not been quantified, but which is consistently observed, is a muscle trembling that is readily discerned when a *frogleg* animal is held in the hand. In addition, some *frogleg* animals were observed to be susceptible to a type of seizure, especially if startled by sudden noise or other disruption in the animal room. The *Bckdk* knockout mouse is also prone to seizures [2], and human patients with mutations in *Bckdk* have been described as having symptoms of autism and epilepsy, with developmental delays, neurobehavioral abnormalities, and intellectual disability [3, 13]. This is also true in some other mouse models of autism spectrum disorders [14, 15], as well as in rats fed an isoleucine-deficient diet and exposed to pentylenetetrazole [16].

Since the hind limb abnormality is the dominant feature of the *frogleg* phenotype, we have evaluated the structure and function of neuromuscular systems in the hind limbs. Histological studies revealed only a very low frequency of myelination defects in selected nerves and scattered abnormal muscle fibers suggestive of denervation atrophy. For example, the sciatic nerve in the *frogleg* animals was found to have scattered axons that were thinly myelinated and others showing evidence of active demyelination. These changes may explain in part the decreased sciatic CMAP amplitude, which is a function of the number of functional axons or fibers; however, the sharp decrease in amplitude is out of proportion to the small number of obviously affected axons. Neuromuscular junctions in the hind limb were also examined on both the soleus muscle, a slow twitch or postural muscle, and the EDL, a fast twitch muscle. Progressive and marked denervation was found to occur in the soleus, with less than 5% of junctions remaining intact by 7 months of age. In contrast, at 7 months the EDL has only a modest decrease in intact junctions, with about 85% still being intact. Completely denervated junctions were not seen in the EDL, but accounted for fully 50% of all junctions evaluated in the soleus at 7 months. Upon analyzing the status of junctions as a function of age for the soleus muscle, we found that at 1 month of age all junctions were intact in the *frogleg* animals. There was a precipitous drop in the number of intact junctions between one and two months of age for the *frogleg* rats, with 50% of the junctions being fully or partially denervated. Thereafter, the loss of fully innervated junctions became a more gradual process until only about 5% of total junctions were intact at 7 months. Clearly, the loss of functional neuromuscular junctions on the soleus muscle is not a primary cause of the *frogleg* hind limb defect since that defect is present before there is any loss in intact junctions. It is possible that early on there is a physiological defect in NMJ transmission, with loss of NMJ innervation occurring later.

There is a range of severity in all these phenotypic characteristics within the *frogleg* population, both in males and females; however, males are generally more severely affected than females. The failure of the *frogleg* animals to reproduce is probably explained in the males by defective spermatogenesis, but in the females we have no explanation at this time. Knockout mice also showed decreased fertility [2]. There are clearly CNS effects of the *frogleg* mutation. This is obvious from the markedly decreased brain weight. Brain weight was also reduced in the knockout mice [2], and microcephaly is present in some human patients [13]. Brain MRI analyses done in living animals confirm decreased brain volume and greatly enlarged ventricles in the *frogleg* homozygotes. It is likely that the muscle trembling and seizures observed in *frogleg* rats are manifestations of abnormal brain function. Still, cellular abnormalities observed in histological analyses of *frogleg* brain sections were minimal, as was also reported to be the case in the knockout mice [2].

While it is apparent that the mutation in *Bckdk* is ultimately responsible for *frogleg*, the molecular basis for the various abnormalities associated with the phenotype is unknown. This is also true for the *Bckdk* knockout mouse and the human subjects with mutations in *Bckdk*. Several possibilities exist: first, BCAAs are necessary building blocks for all proteins, and reduced levels may impair protein synthesis, either generally or in certain tissues or under particular conditions. It has been suggested that this could explain deficits in growth or in brain weight [1]. Secondly, leucine may be of special significance, because it can act through the mechanistic Target of Rapamycin (mTOR) signaling pathway to stimulate translation, promote insulin release and inhibit protein degradation by autophagy [5]. Unlike most essential amino acids which are metabolized just in the liver, the metabolism of BCAAs occurs in many tissues including the CNS [1]. In the brain the BCAAs, and particularly leucine which enters from the blood more rapidly than other amino acids, are a critical source of amino groups required for the synthesis of glutamate by neurons [17]. Glutamate serves as both a neurotransmitter and a critical metabolic intermediate in the CNS and it is plausible that disruption of glutamate balance could play a major role in the neurological effects seen in *frogleg*.

In conclusion, the complex phenotype seen in the *frogleg* rat arises from a mutation in the gene *Bckdk* that causes marked decrease in the circulating levels of the essential amino acids leucine, isoleucine and valine. A variety of mechanisms could be involved in how reduced BCAA levels lead to the abnormalities observed in the various affected tissues. Thus, further study of this unique rat strain, *Bckdk*^*frogleg/frogleg*^ may help us to better understand the function of BCAAs in health and disease, particularly of the nervous system.

## Materials and Methods

### Animals

The *frogleg* phenotype arose in a breeding colony of Sprague-Dawley rats which had originated with animals purchased from Taconic Farms (Hamilton, NY). Additional wild type Sprague-Dawley NTac:SD (Taconic Farms) and Lewis LEW/SsNHsd (Harlan, Indianapolis, IN) rats were introduced into the colony for breeding purposes, as outlined below. Since we have been unsuccessful in getting animals with the *frogleg* phenotype to reproduce, the phenotype was perpetuated by mating *frogleg* heterozygotes with each other. The *frogleg* rats, which require no special husbandry, were bred and maintained at Spring Valley Laboratories, Inc (Woodbine, MD), an AAALAC accredited animal facility. Specific pathogen free (SPF) animals were obtained initially, and they were maintained in SPF conditions with regular sentinel surveillance. They were housed on contact bedding and fed Teklad 2018 rodent chow ad libitum, with a 12:12 light cycle. All animal procedures were designed to minimize any pain or distress for the animals and were approved by the Animal Care and Use Committees of Johns Hopkins University and Spring Valley Laboratories.

### Linkage Analysis and Genotyping

To establish an initial gene linkage population, two normal Lewis females were obtained from Harlan Sprague-Dawley (Indiananapolis, IN) and were mated to 2 Sprague-Dawley males that had been proven to be heterozygous for the *frogleg* trait through mating studies. Six male and 9 female progeny were obtained from these matings, 50% of which were expected to be heterozygous for *frogleg*. Random matings of these F1 animals ultimately revealed 2 mating pairs that produced litters that included *frogleg* progeny. From several litters of the F2 generation obtained from these matings, 21 pups were chosen (10 *frogleg* and 11 unaffected). These animals were euthanized and genomic DNA extracted from their livers (DNeasy Kit, Qiagen). Similarly, one of the parental pairs and a normal Lewis rat were euthanized and DNA extracted. These 24 samples constituted the first set of genotyping samples. Gene linkage analysis was performed essentially as reported earlier [9] for the *Nuc1* mutation, using the fluorescently labeled Rat Map Pairs screening set (Invitrogen). After the gene was localized to a region of rat chromosome 1, additional markers were obtained and used to reduce the interval.

### Whole Genome Sequencing

DNA was extracted from livers of a *frogleg* homozygous mutant and a wild-type littermate by standard protocols. Size selected genomic fragment libraries were prepared using the IonXpress Plus fragment library kit according to the manufacturer’s protocol, with enzymatic fragmentation performed for 12 ½ minutes at 37°C and size selection performed on a Pippen Prep set to collect fragments in the 280-342bp range. Libraries were quantitated by qPCR using the Ion Library Quantitation kit (Thermo Fisher Scientific), templated using the Ion PI template OT2 200 Kitv2 and whole genome sequencing performed on the Ion Proton platform (Thermo Fisher Scientific) using PI v2 chips and Ion PI sequencing 200 chemistry. Combining data from 3 runs for each sample the *frogleg* mutant and WT samples were sequenced to a depth of 10.4x and 11.8x respectively. The reads were mapped to UCSC rn5 with tmap-f3, and variants called with the TVC plugin in Torrent Suite. The genotypes at the variant positions were called by a pipeline consisting of mpileup function within samtools (version 1.2) along with calls function and vcfutils.pl in bcftools (version 1.2). AB genotypes were generated by parsing the counts in the VCF files. The variant functional annotation was performed with an in-house tool (SNP.to.ucsc-functions.pl) and Ensembl's VEP.

### Molecular modeling

Protein sequence of mitochondrial BCKDK (alternate name: 3-methyl-2-oxobutanoate dehydrogenase kinase) from rat was obtained from the UniProt database (http://www.uniprot.org, Acc #Q00972). Crystal structures of rat BCKDK were selected from Protein databank (PDB), files = 3TZ4 (residues 68–405), 4DZY (67–405), and 4H7Q (residues 74–405) and used as the templates. A high quality hybrid structure of the rat BCKDK was generated using molecular visualization and molecular dynamics program Yasara (www.yasara.org). The same program was used to generate the G369E mutant variant and for refinement of protein structures using 7.5 ns molecular dynamics in water.

### Preparation and Analysis of Brain Lysates

Anterior-posterior slices of brain tissue taken near the midline from freshly euthanized rats, were weighed and homogenized at an approximate concentration of 600 mg wet tissue weight per ml in cold RIPA buffer (Millipore) containing protease inhibitor cocktail (Sigma, P-8340). After homogenization in a ground glass tissue grinder, an aliquot of the homogenate was removed and to the remaining portion of homogenate was added phosphatase inhibitor cocktail 3 (Sigma, P-0044). Each sample was then put into an Eppendorf tube and placed on an end-over-end roller for 30 minutes at 4°C. The samples were then centrifuged at 16,000xg for 30 minutes at 4°C and supernatants collected for analysis. To the aliquots lacking phosphatase inhibitors, Calf Intestinal Alkaline Phosphatase (Promega, M1821) was added according to the manufacturer’s instructions and incubated for 30 minutes at 37°C. Aliquots of the samples were prepared for SDS-PAGE and loaded onto 4–12% Bis-Tris minigels (Novex). After electrophoresis, gels were either stained with colloidal Coomassie blue or transferred to nitrocellulose using the iBlot system (Invitrogen) for western blotting. Primary antibodies used were from Abcam: anti-BCKDH (ab138460) and anti-BCKDH phospho S293 (ab200577). The secondary antibody was goat anti-rabbit IgG labeled with horseradish peroxidase (Kirkegaard and Perry) and detection was with ECL (GE Healthcare).

### Amino Acid Analysis

Amino acid analysis was performed by standard ion exchange chromatography procedures using a Biochrom 30+ amino acid analyzer at the Kennedy Krieger Institute Biochemical Genetics Laboratory.

### Pathology

1. **Morphology and Histopathology.** *Frogleg* and wild type control rats were compared by survey radiography, as well as complete macroscopic and histological survey analyses. These studies were performed at the Johns Hopkins Phenotyping Core Facility using their standard protocols, as described previously [18]. Macroscopic examination included observation of gait and stance, and confirmation of *frogleg* phenotype. After carbon dioxide euthanasia, blood was drawn for hematology and serum saved, and a complete *post mortem* examination was performed. Brains, livers, hearts, spleens and kidneys were weighed, and more than 40 tissues collected for immersion fixation in 10% neutral buffered formalin. Heads, spine, and limbs were fixed and decalcified in Formical-4 ^™^ fixative decalcifying solution (Decal Chemical Corp, Tallman, NY). The fixed tissues were processed and embedded in paraffin, sectioned at 4μ, and stained with hematoxylin and eosin, as described previously [18]. Central nervous system (CNS) sections were stained with Luxol Fast Blue (LFB), and Periodic Acid Schiff (PAS) stains. Representative sections of brain, nerve, muscle, and spleen were scanned digitally for virtual microscopy and morphometry using the Zeiss Mirax system, Carl Zeiss, Inc.
2. **Magnetic Resonance Imaging (MRI).** Rats were anesthetized with isoflurane and then the brains were imaged with a 4.7 Tesla nuclear magnetic resonance scanner (Bruker Biospin, Billerica, MA). Fast spin echo sequence was used for T2 weighted imaging with the following parameters: TR = 700 ms and effective TE = 63.7 ms, echo train length = 8. Multiple slice two dimensional images were acquired with in plane imaging matrix 212x176 and field of view 30x27 mm. Thickness of slices was 0.7 mm, without a gap between slices. 42 slices were imaged, covering the entire brain. The imaging resolution was 0.14 x 0.15 x 0.7 mm. After zero padding, the image resolution was 0.12 x 0.11 x 0.7 mm. Using the acquired MRI data, total brain volume and the volume of the lateral ventricles were calculated.
3. **Electrophysiological Analysis.** Wildtype and *frogleg* rats (10 weeks and 7 months old) were anesthetized with chloral hydrate i.p. using a sterile 25 gauge needle and placed on a heating pad during the entire procedure to maintain body temperature. Using sterile monopolar needle electrodes, nerve conduction studies were performed with a stimulating electrode at the sciatic notch and the reference approximately 0.5 cm from that site. The recording electrodes were placed in the plantar surface of the foot. The ground electrode was placed in the tail. The pulse stimulus was 0.5 ms with a supramaximal stimulus of 6-10V. Electrophysiological recordings across the nerve segment were made using an ADI (Greenwich, CT) Powerlab 8SP stimulator and BioAMP amplifier followed by computer assisted data analysis (Scope 3.5.6; ADI). The distal motor latency of the compound muscle action potentials (CMAP) was measured in milliseconds. This includes the duration of motor nerve conduction between the stimulating and recording electrodes plus the time of synaptic transmission. Ten tracings were recorded from each tibial nerve in order to establish reproducibility of the response obtained. The average of these tracings was then incorporated into the data analysis. These studies were performed bilaterally. Following completion of the study, animals were placed in a cage on a heating pad until they recovered from anesthesia. Animals were observed for a period of 4 hours after emergence from anesthesia.
4. **Transmission electron microscopy (TEM).** Samples for TEM were obtained from *frogleg* and control animals that were deeply anesthetized, then perfused, first with PBS and then with 4% paraformaldehyde. Sections of sciatic and sural nerves were dissected and post-fixed in 4% paraformaldehyde-3% glutaraldehyde. To distinguish if the mutation affects sensory or motor pathways or both, we also isolated L4 and L5 ventral roots for motor neurons and L4 and L5 dorsal roots for sensory neurons. Samples were imbedded in epon and ultrathin sections were cut for analysis.
5. **Analysis of neuromuscular junctions.** To evaluate neuromuscular junctions in the hindlimb, the soleus and extensor digitorum longus (EDL) muscles were dissected from freshly euthanized *frogleg* and wild type rats and processed as previously described [19]. The isolated muscles were pinned in a small dish and post-fixed for 20 minutes in 4% paraformaldehyde, then washed at room temperature 3 times for 10 minutes each in 1X PBS (pH 7.4). The washed muscles were treated for 30 minutes in 0.1M glycine and then were treated for 15 minutes with rhodamine-conjugated alpha-bungarotoxin (Invitrogen) diluted 1:200 in 1X PBS to label acetylcholine receptors (AChRs) in the muscle. After 3 washes with PBS as above, the muscles were placed in pre-cooled methanol for 7 minutes in the -20°C freezer, then washed in 1X PBS 3 times for 10 minutes each. The muscles were then blocked for 1 hr at room temperature in 0.2% TBP (PBS containing 0.2% Triton and 2% bovine serum albumin). They were then incubated overnight at 4°C in 0.2% TBP containing primary antibodies. The antibodies used were a mouse monoclonal against synaptic vesicle glycoprotein 2A (Sv2, DSHB Cat No. SV2-s) at 1:10 dilution and a mouse monoclonal against neurofilament (SMI312R, Covance) at 1:1000. Samples were then rinsed at least 3 times in PBS as above, and placed in 0.2% TBP containing the secondary antibody (FITC goat anti-mouse IgG1, Jackson Immuno) at 1:200 dilution for 1 hr at room temperature. After final rinses (3X10 minutes) in PBS, the muscles were cleaned of any residual connective tissue, the top layer was excised and then mounted on slides using Vectashield (Vector Labs). The slides were examined by immunofluorescence and publication images acquired using a Zeiss LS 150 confocal microscope. A minimum of 100 junctions was evaluated for each muscle analyzed, with a minimum of 3 muscles from different animals used for each data point. Labeled muscles were visualized, quantified and categorized as (1) intact (normally innervated by the original axon), (2) completely denervated (no occupation of AChRs by nerve terminal) or (3) partially denervated (partial occupation of AChRs by nerve terminal).

### Statistical Analysis

Statistical analysis was performed using Microsoft Excel and GraphPad 5.0 software. The P values were determined by two-tailed Student’s *t*-test. Significance was defined as P<0.05. Data are reported as mean +/- S.D. or SEM. Details regarding specific analyses are provided in the figure legends where relevant.

## Supporting Information

S1 Table A complete list of the homozygous variants found in the *frogleg* interval. (XLSX)

S2 Table Putative functional variants present in the *frogleg* interval and their possible consequences. (XLSX)

S3 Table Complete plasma amino acid data in μmol/l for 2 groups of rats. Animals in group 1 are from a single litter 6 weeks old. The animals in Group 2 are from a litter 8 weeks old. The genotype of each animal is indicated at the top (WT = wild type; FL = *frogleg*; HET = heterozygote for frogleg). Data for branched-chain amino acids is highlighted (yellow for WT and HET; green for FL).
(XLSX)

S1 Video A *frogleg* homozygous rat and an unaffected littermate are viewed as they move about the cage. Note the splayed hind limbs and abnormal gait of the *frogleg* animal.
(MP4)

S2 Video Weanling *frogleg* and wild type littermates are shown suspended by the tail. Note the clenching and obvious tremor of the hind limbs of the *frogleg* as compared to its normal littermate.
(MP4)
